# Diagnostic accuracy of NT-proBNP to predict the incidence of CSA-AKI: A systematic review and meta-analysis

**DOI:** 10.1097/MD.0000000000039479

**Published:** 2024-10-25

**Authors:** Jiaying Zhang, Xin Li, Xiaofeng Yu

**Affiliations:** a Department of Nephrology, The Third Hospital of Mianyang/Sichuan Mental Health Center, Mianyang, Sichuan, China; b Department of Neurosurgery, Chengdu Third People’s Hospital, Chengdu, Sichuan, China; c Department of Cardiology, The Third Hospital of Mianyang/Sichuan Mental Health Center, Mianyang, Sichuan, China.

**Keywords:** acute kidney injury, cardiac surgery, meta-analysis, NT-proBNP

## Abstract

**Background:**

Cardiac surgery–associated acute kidney injury (CSA-AKI) is a severe complication following cardiac surgery. Early identification and diagnosis are critical. In this study, we aim to systematically assess the diagnostic value of N-terminal pro-B-type natriuretic peptide (NT-proBNP) for CSA-AKI.

**Methods:**

The PubMed, Embase, Web of Science, and Cochrane Library databases were searched from January 1971 to October 2023 to identify prospective observational and retrospective observational studies. Data extraction and study screening were carried out independently by 2 authors. The methodological quality of the included studies was evaluated by the Quality Assessment of Diagnostic Accuracy Studies 2 standards, and all statistical analyses were conducted by Stata 15.0.

**Results:**

Seven studies including 37,200 patients were identified. The pooled sensitivity of 0.67 (95% credible interval [CI] = 0.56–0.77), specificity of 0.55 (95% CI = 0.45–0.64), area under the summary receiver operating characteristic curve of 0.65 (95% CI = 0.60–0.69), positive likelihood ratio of 1.5 (95% CI = 1.2–1.8), negative likelihood ratio of 0.60 (95% CI = 0.46–0.79), and diagnostic odds ratio of 2 (95% CI = 2–4) suggested that patients with higher preoperative NT-proBNP levels (pg/mL) are at higher risk of developing acute kidney injury after cardiac surgery. However, NT-proBNP lacks sufficient sensitivity and specificity to reliably predict CSA-AKI.

**Conclusion:**

Our findings suggest that the diagnostic accuracy of NT-proBNP to predict the incidence of CSA-AKI is limited. However, we provide novel perspectives on the early detection of CSA-AKI biomarkers, and it is urgent to identify more precise and practical biomarkers for the early diagnosis of CSA-AKI.

## 1. Introduction

Acute kidney injury (AKI) is a severe complication after cardiac surgery, which not only has a high morbidity and mortality but also brings a heavy economic burden to hospitalized patients. Every year, >2 million cardiac surgeries are conducted worldwide.^[1]^ The morbidity of cardiac surgery–associated acute kidney injury (CSA-AKI) is 2% to 50%,^[2]^ with a mortality ranging from 3.8% to 54.4%^[3]^; around 1% to 15% of patients with CSA-AKI require renal replacement therapy.^[4]^ The pathological and physiological mechanisms of CSA-AKI are complex, mainly including ischemia-reperfusion injury, renal hypoperfusion, inflammation, oxidative stress, neurohormonal activation, nephrotoxic exposure, raised intra-abdominal pressure, renal congestion, and cardiopulmonary bypass.^[2,5,6]^ The Risk, Injury, Failure, Loss, and End-stage renal disease criteria,^[7]^ Acute Kidney Injury Network criteria,^[8]^ and Kidney Disease Improving Global Outcomes criteria^[9]^ are the primary diagnostic criteria for CSA-AKI. These criteria are based on traditional markers of renal function, serum creatinine, and urinary output. Due to the kidneys’ strong reserve capacity, structural and functional damage frequently occurs before the increase of urea nitrogen and creatinine; hence, these indicators are not ideal as sensitive biomarkers for predicting the incidence of CSA-AKI.^[10]^ Therefore, finding a sensitive biomarker for early identification of CSA-AKI is significant.

The brain natriuretic peptide (BNP) is a hormone that is synthesized and secreted by myocardial cells.^[11]^ Initially, pro-BNP, composed of 108 amino acid residues, is synthesized by myocardial cells. It is subsequently cleaved 1:1 into biologically active BNP and nonbiologically active N-terminal pro-B-type natriuretic peptide (NT-proBNP).^[12,13]^ With its 32 amino acid residues, BNP binds to the blood’s natriuretic peptide receptor A to exert the effect of diuresis, vasodilatation, inhibition of renin and aldosterone production, and cardiac and vascular myocyte growth. NT-proBNP is an N-terminal amino acid residue composed of 76 amino acid residues. Compared to BNP, it has no biological activity.^[11]^ Furthermore, NT-proBNP levels have been reported to be elevated in AKI patients,^[14,15]^ particularly in those after cardiac surgery, according to some studies.^[16,17]^

To fully understand the correlation between elevated levels of NT-proBNP and CSA-AKI, we performed this meta-analysis to assess the diagnostic value of NT-proBNP for CSA-AKI and also hope that this study can provide evidence-based medical guidance for the early clinical prediction of CSA-AKI.

## 2. Methods

### 2.1. Search strategy

The search databases for this study were PubMed, Embase, Web of Science, and Cochrane Library from January 1971 to October 2023. The following keywords were used: acute kidney injury or acute kidney injuries or kidney injuries, acute or kidney injury, acute or acute renal injury or acute renal injuries or renal injuries, acute or renal injury, acute or renal insufficiency, acute or acute renal insufficiencies or renal insufficiencies, acute or acute renal insufficiency or kidney insufficiency, acute or acute kidney insufficiencies or kidney insufficiencies, acute or acute kidney insufficiency or kidney failure, acute or acute kidney failures or kidney failures, acute or acute renal failure or acute renal failures or renal failures, acute or renal failure, acute or acute kidney failure and cardiac surgery or cardiac surgical procedures or cardiac operation or cardio surgery or cardiopulmonary bypass and pro-brain natriuretic peptide or N-terminal pro-BNP or NTproBNP or N-BNP peptide or NT-BNP or amino-terminal pro-brain natriuretic peptide or aminoterminal pro-B-type natriuretic peptide or NT-proBNP or proBNP(1-76). The detailed search strategy is shown in Appendix 1, Supplemental Digital Content, http://links.lww.com/MD/N461. The present study has been registered on PROSPERO (international prospective register of systematic reviews) (registration No. CRD42023473949).

### 2.2. Study selection

The inclusion criteria were as follows: articles investigated the association between NT-proBNP and the incidence of CSA-AKI; the study population included patients ≥18 years old undergoing cardiac surgery; a diagnostic 4-grid table could be constructed; CSA-AKI was clearly defined; the study types were prospective observational studies and retrospective observational studies; the full text of the study was available in English.

The exclusion criteria were as follows: review articles, letters, comments, or opinions; in vitro or animal experiments; studies from which the diagnostic 4-grid table could not be directly or indirectly extracted.

Two authors (JZ and XL) independently assessed the selected studies for the final analysis, and any discrepancies were resolved through consultation with the third author (XY).

### 2.3. Data extraction

Two authors (JZ and XL) extracted the following data by using standardized forms: date of publication, first author, country, age, sex, number of included patients, patient types, study types, outcomes of patients, definition of CSA-AKI, sample, specimen sampling time, and *P* value (Table 1), measurement method of NT-proBNP, cutoff points, area under the curve, true positives, true negatives, false positives, false negatives, sensitivity (SEN), and specificity (SPE) (Table 2).

**Table 1:** The basic information table of included studies.

Study	Country	Mean age		Male (%)		Number of included patients		Patient types	Study types	Outcomes	Definition of AKI	Specimen sampling time	Sample	*P* value
		AKI	No AKI	AKI	No AKI	AKI	No AKI							
Ballotta et al, 2010^[18]^	Italy	NA	NA	NA	NA	7	24	CABG	Retrospective cohort study	AKI	Peak postoperative serum creatinine value 2 times the preoperative value and >2.0 mg/dL	Before surgery	Plasma	.032
Belley-Côté et al, 2016^[19]^	North America	70.7 ± 10.4	71.53 ± 10	27 (73%)	628 (68%)	37	923	CABG or valve	Prospective cohort study	AKI	Doubling in serum creatinine from baseline or requiring renal replacement therapy during hospital stay	Before surgery	Plasma	.03
Elíasdóttir et al, 2008^[20]^	Iceland	NA	NA	NA	NA	16	119	CABG or OPCAB or both	Retrospective cohort study	AKI	>50% increase in serum creatinine levels after surgery compared with preoperative levels	Before surgery	Serum	<.001
MacMillan et al, 2022^[21]^	Canada	67.9 ± 10.6	66.0 ± 10.1	58 (68%)	56 (66%)	49	49	Cardiac surgery	Prospective nested case-control study	AKI	>50% relative or >26 µmol/L absolute rise in serum creatinine above preoperative value within 48 h of surgery or new-onset dialysis within 48 h of surgery	Before surgery	Plasma	.78
Zelt et al, 2018^[22]^	Canada	67.1* (61.1–73.2)	66.0* (63.8–68.2)	11 (61.1%)	61 (68.5%)	11	89	Major elective cardiac surgery requiring CPB	Prospective nested case-control study	AKI	>50% relative or >26 µmol/L absolute rise in serum creatinine above preoperative value within 48 h of surgery or new-onset dialysis within 48 h of surgery	Before surgery	Plasma	.12
Verwijmeren et al, 2021^[23]^	The Netherlands	75* (72–78)	74* (72–77)	57 (64.8%)	301 (66.7%)	88	451	Cardiac surgery	Prospective cohort study	AKI	Absolute increase of serum creatinine ≥26.5 µmol/L within 48 h after surgery or 1.5 times the baseline value within the first 5 d	Before surgery	Plasma	.005
Wang et al, 2021^[17]^	China	60 ± 11	57 ± 12	7927 (66.1%)	15,405 (66.0%)	11,999	23,338	Cardiac surgery	Retrospective cohort study	AKI	Serum creatinine increase of 26 µmol/L within a 48-h window or a 1.5–1.99 increase within the initial 7 d	Hospital admission	Plasma	<.001

AKI = acute kidney injury, CABG = coronary artery bypass grafting, CPB = cardiopulmonary bypass, NA = not available, OPCAB = off-pump coronary artery bypass.

* Median (5th to 95th percentile ranges).

**Table 2:** The information of 4-fold table.

Study	Assay	Cutoff (pg/mL)	AUC	SEN%	SPE%	TP/FP/TN/FN
Ballotta et al, 2010^[18]^	NA	1304	0.84	71%	67%	5/8/16/2
Belley-Côté et al, 2016^[19]^	Roche Diagnostics	504	0.64	78%	50%	29/447/447/8
Elíasdóttir et al, 2008^[20]^	Electrochemiluminescence immunoassay (Roche Diagnostics)	404	0.86	88%	70%	14/36/83/2
MacMillan et al, 2022^[21]^	Electrochemiluminescence immunoassays with a Roche Cobas e411 analyzer	459	0.55	49%	69%	24/15/34/25
Zelt et al, 2018^[22]^	Electrochemiluminescence immunoassays with a Roche Cobas e411 analyzer.	476	0.74	77%	72%	14/25/64/4
Verwijmeren et al, 2021^[23]^	Cobas 8000 platform (Roche Diagnostics)	486 for women 470 for men	0.75	65%	55%	57/205/246/31
Wang et al, 2021^[17]^	Elecsys proBNP, Cobas E analyzer (Roche Diagnostics)	Isolated CABG = 245	NA	62%	52%	3284/6029/6616/1988
		Isolated valve surgery = 365		72%	44%	2174/2806/2209/844
		Concomitant CABG + valve surgery = 735		57%	58%	584/362/493/446
		Septal myectomy = 493		89%	24%	222/450/145/27

AUC = area under the summary receiver operating characteristic curve, CABG = coronary artery bypass grafting, FN = false negative, FP = false positive, NA = not available, SEN = sensitivity, SPE = specificity, TN = true negative, TP = true positive.

### 2.4. Quality assessment

We evaluated these studies according to the Quality Assessment of Diagnostic Accuracy Studies 2 standards in 4 domains: patient selection, index test, reference standard, and flow and test timing (Figure S1, Supplemental Digital Content, http://links.lww.com/MD/N462). Any disagreements were resolved by discussion with a third reviewer (XY).

### 2.5. Statistical analysis

Stata 15.0 was used for this meta-analysis. A bivariate random-effects regression model was performed to calculate SEN, SPE, positive likelihood ratio, negative likelihood ratio, diagnostic odds ratio, and the corresponding 95% credible interval (CI). A summary receiver operating characteristic (SROC) curve was drawn to assess the overall diagnostic accuracy. To assess the heterogeneity, I^2^ statistics were used, with values exceeding 50% indicating substantial heterogeneity. The Deek funnel plot was adopted to assess publication bias. To assess clinical applications, we created a Fagan nomograph and likelihood ratio scattergram. In this study, *P* < .05 indicated that the difference was statistically significant.

### 2.6. Ethical statement

All analyses were based on previous published studies; thus no ethical approval and patient consent are required.

## 3. Results

### 3.1. Study inclusion

**Figure 1.**
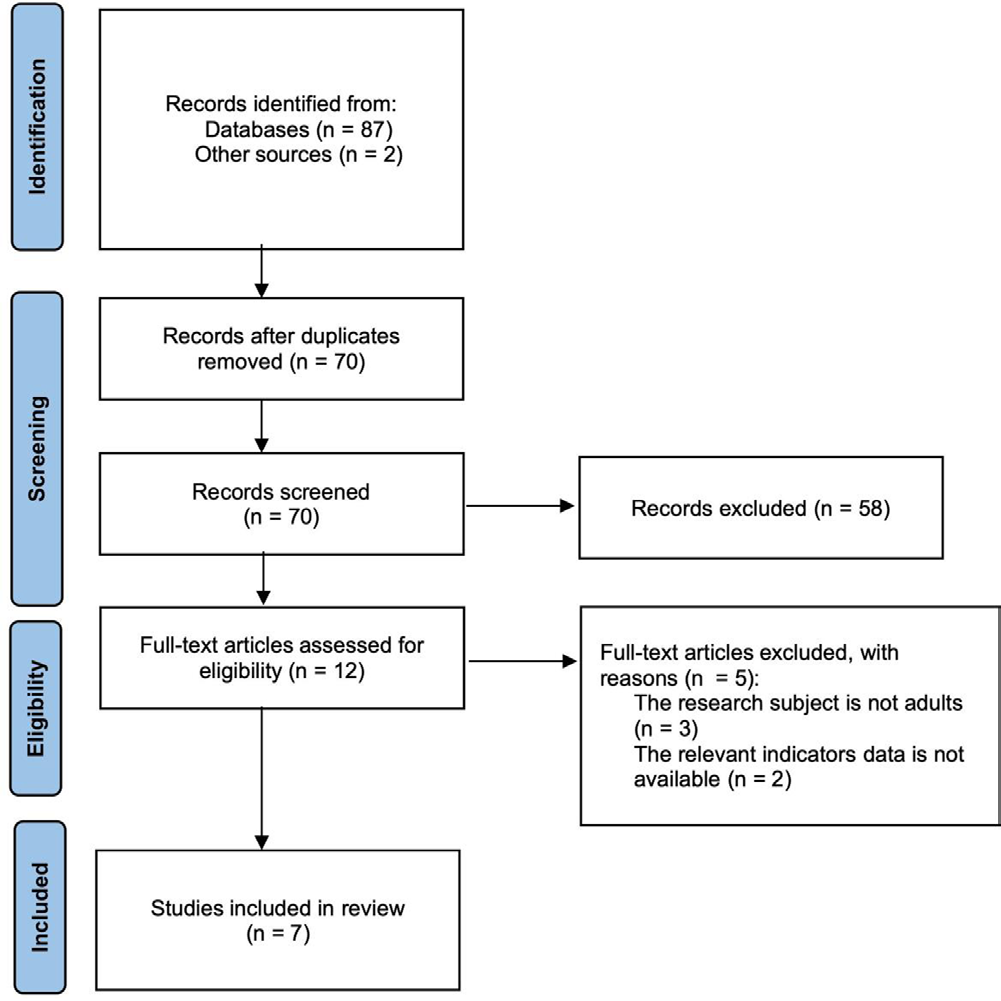
Flowchart of study selection.

Figure 1 depicts the study selection procedure. As a result of the literature search, 89 studies were identified, of which 19 duplicate publications were excluded. After removing duplicates, 70 publications remained, of which 58 were excluded after the title and abstract reading. The remaining 12 articles were further scrutinized by reading the full text. Three studies were excluded due to the research subjects not being adults; 2 studies were excluded because the relevant indicators were not available. In total, 7 studies, including 37,200 patients, fulfilled the inclusion criteria and were ultimately included in this meta-analysis (Table 1).

Seven studies were included (4 prospective and 3 retrospective), with patients from 6 different nations and regions (2 from Canada, 1 from North America, 1 from China, 1 from Italy, 1 from the Netherlands, and 1 from Iceland) that were published between 2008 and 2022 (Table 1).

### 3.2. Study quality and publication bias

**Figure 2.**
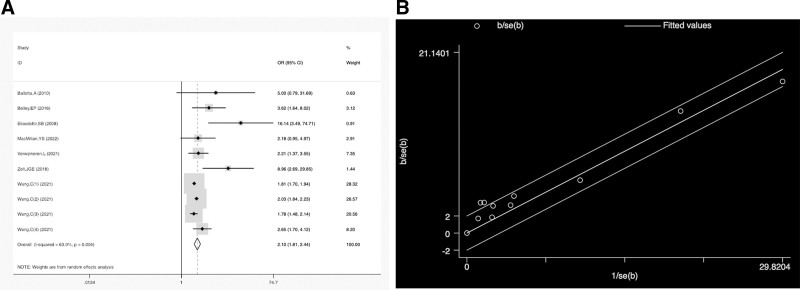
(A) The result of heterogeneity. (B) Galbraith plot for heterogeneity. OR = odds ratio.

In this meta-analysis, the heterogeneity I^2^ was 0.63 (Fig. 2A), and the Galbraith plot revealed that the studies by Elíasdóttir et al and Zelt et al might be the main sources of heterogeneity (Fig. 2B).

**Figure 3.**
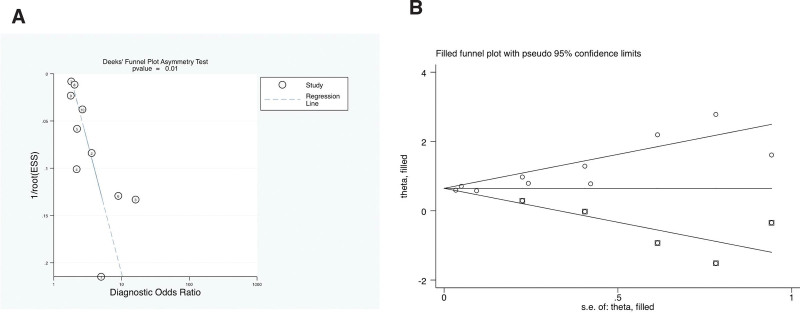
(A) Deek funnel plot asymmetry test for publication bias. (B) Filled funnel plot for publication bias. ESS = effective sample size.

Figure 3 shows the risk of bias in the 7 included studies. The result of Deek funnel plot was significant for the outcome (*P *= .01), which was in accordance with the asymmetrical appearance of the drawn funnel plot (Fig. 3A). To compensate for the effect of the putative missing studies, trim and fill analysis was performed (Fig. 3B). After inserting 5 imputed studies, the results showed a heterogeneity test: Q = 44.718, *P* < .001, using a random-effects model. The combined effect indicator was logOR of 0.647, with a 95% CI of 0.477–0.817. Accordingly, after adding 5 studies, the results still have statistical significance, so the combined results are robust.

### 3.3. Diagnostic value of NT-proBNP for CSA-AKI prediction

**Figure 4.**
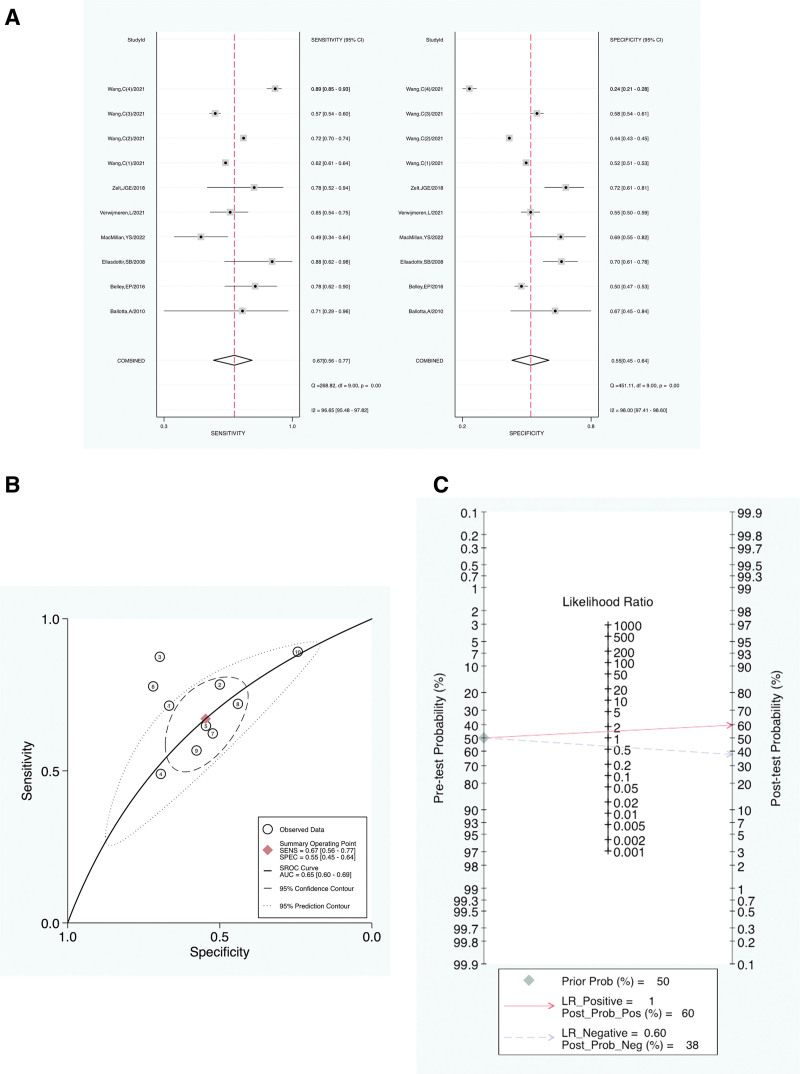
(A) Forest plot of the SENS and SPEC of NT-proBNP for the diagnosis of CSA-AKI. (B) Forest plot of the pooled positive LR and the pooled negative LR of NT-proBNP for the diagnosis of CSA-AKI. (C) SROC for the included studies. (D) Fagan nomogram of NT-proBNP for the diagnosis of CSA-AKI. AUC = area under the summary receiver operating characteristic curve, CSA-AKI = cardiac surgery–associated acute kidney injury, DLR = diagnostic likelihood ratio, LR = likelihood ratio, Neg = negative, NT-proBNP = N-terminal pro-B-type natriuretic peptide, Prob = probability, Pos = positive, SENS = sensitivity, SPEC = specificity, SROC = summary receiver operating characteristic curve.

The pooled SEN of NT-proBNP in predicting CSA-AKI was 0.67 (95% CI = 0.56–0.77); the pooled specificity was 0.55 (95% CI = 0.45–0.64); the pooled predictive odds ratio was 2.46 (95% CI = 1.60–3.79); the pooled positive likelihood ratio was 1.48 (95% CI = 1.23–1.78); the pooled negative likelihood ratio was 0.60 (95% CI = 0.46–0.79); and the area under the SROC curve was 0.65 (95% CI = 0.60–0.69). The SEN, specificity, pooled positive likelihood ratio, pooled negative likelihood ratio, and SROC curve are presented in Fig. 4A–C.

Fagan plot was employed to reflect the clinical applicability of NT-proBNP in predicting CSA-AKI. The incidence of CSA-AKI varies significantly in different contexts. It is estimated to range from 2% to 50%. Therefore, assuming a prior probability of 20%, the probability of the diagnosis of CSA-AKI is 0.27 in the case of a positive likelihood ratio of 1, and the probability of the diagnosis of no CSA-AKI is 0.13 in the case of a negative likelihood ratio of 0.60. The clinical application is shown in Figure 4D.

According to the findings above, we found that patients with higher preoperative NT-proBNP levels (pg/mL) are at higher risk of developing AKI after cardiac surgery. However, NT-proBNP lacks sufficient SEN and SPE to reliably predict CSA-AKI.

## 4. Discussion

After applying the inclusion and exclusion criteria, 7 articles were included in this study to evaluate the value of NT-proBNP for CSA-AKI. Meta-analysis revealed the level of preoperative NT-proBNP (pg/mL) was higher in patients with AKI than in patients without AKI after cardiac surgery. However, NT-proBNP lacks sufficient SEN and SPE to reliably predict CSA-AKI (area under the SROC = 0.65, SEN = 0.67, and SPE = 0.55).

AKI is a common and serious complication after cardiac surgery. According to recent studies, CSA-AKI is strongly associated with adverse clinical outcomes, such as the increase of perioperative mortality, the prolongation of intensive care unit and hospitalization residence time, and the increase of treatment cost.^[24]^ Early identification and diagnosis of AKI are crucial, as prevention before clinical presentation may be more effective than treating patients with confirmed AKI. However, the current diagnostic criteria are not suitable for timely diagnosis of AKI.^[25]^ Therefore, the discovery of novel biomarkers is of great significance for the early diagnosis of CSA-AKI.

In this study, we concluded that the level of preoperative NT-proBNP (pg/mL) was higher in patients with AKI than in patients without AKI after cardiac surgery. The following reasons might be involved to some extent. First, NT-proBNP is mostly eliminated by the kidneys and has a steady property with a relatively long half-life. Thus, in the setting of cardiac surgery, hemodynamic impairment may reduce the clearance of NT-proBNP.^[26]^ Second, systematic vasodilation and antagonism of the renin-angiotensin-aldosterone system mediated by NT-proBNP might directly lead to renal hypoperfusion and cause renal injury.^[17]^ Furthermore, NT-proBNP may be an indicator of elevated inflammation following heart surgery, which is crucial for the emergence and progression of CSA-AKI.^[27]^ However, further investigation is required to determine the possible mechanism underlying the interaction between CSA-AKI and NT-proBNP.

Nevertheless, NT-proBNP lacks sufficient SEN and SPE to reliably predict CSA-AKI. The 3 commonly used scoring systems to predict postoperative AKI and receipt of renal replacement therapy are the Cleveland Clinic model, the Mehta score, and the Simplified Renal Index. The main scoring content includes New York Heart Association class, diabetes, preoperative renal function, chronic obstructive pulmonary disease, left ventricular ejection fraction, myocardial infarction ≤21 days ago, cardiogenic shock, and so on.^[28–30]^ The increase in NT-proBNP levels is associated with all of the above factors; therefore, it lacks sufficient accuracy in predicting CSA-AKI.

Our meta-analysis has various limitations. Initially, there was some discrepancy in the CSA-AKI definition across the included studies. Second, heterogeneity may have resulted from differences in sample size, NT-proBNP assays, cutoff values, and study type across the included studies. Finally, additional subgroup analyses could not be performed to reduce and interpret the heterogeneity because a limited number of studies were included in the meta-analysis.

## 5. Conclusion

Our findings suggest that the diagnostic accuracy of NT-proBNP to predict the incidence of cardiac surgery–associated AKI is limited. However, we provide novel perspectives on the early detection of CSA-AKI biomarkers, and it is urgent to identify more precise and practical biomarkers for the early diagnosis of CSA-AKI.

## Acknowledgments

We appreciate the assistance from The Third Hospital of Mianyang.

## Author contributions

**Methodology:** Jiaying Zhang.

**Writing – original draft:** Jiaying Zhang.

**Conceptualization:** Xin Li.

**Data curation:** Xin Li.

**Formal analysis:** Xin Li.

**Supervision:** Xiaofeng Yu.

**Writing – review & editing:** Xiaofeng Yu.

## Supplementary Material


