# Supplementary material for: Diagnostic accuracy of NT-proBNP to predict the incidence of CSA-AKI: A systematic review and meta-analysis
Source: Medicine (Baltimore). 2024 Oct 25;103(43):e39479. doi: 10.1097/MD.0000000000039479 (PMC11521026; doi:10.1097/MD.0000000000039479)
Supplement: Supplementary file 1 [file medi-103-e39479-s001.docx]

**Pubmed**

1. ((((((((((((((((((((((((((Acute Kidney Injury[MeSH Terms]) OR (Acute Kidney Injuries[Title/Abstract])) OR (Kidney Injuries, Acute[Title/Abstract])) OR (Kidney Injury, Acute[Title/Abstract])) OR (Acute Renal Injury[Title/Abstract])) OR (Acute Renal Injuries[Title/Abstract])) OR (Renal Injuries, Acute[Title/Abstract])) OR (Renal Injury, Acute[Title/Abstract])) OR (Renal Insufficiency, Acute[Title/Abstract])) OR (acute Renal Insufficiencies[Title/Abstract])) OR (Renal Insufficiencies, Acute[Title/Abstract])) OR (Acute Renal Insufficiency[Title/Abstract])) OR (Kidney Insufficiency, Acute[Title/Abstract])) OR (Acute Kidney Insufficiencies[Title/Abstract])) OR (Kidney Insufficiencies, Acute[Title/Abstract])) OR (Acute Kidney Insufficiency[Title/Abstract])) OR (Kidney Failure, Acute[Title/Abstract])) OR (Acute Kidney Failures[Title/Abstract])) OR (Kidney Failures, Acute[Title/Abstract])) OR (Acute Renal Failure[Title/Abstract])) OR (Acute Renal Failures[Title/Abstract])) OR (Renal Failures, Acute[Title/Abstract])) OR (Renal Failure, Acute[Title/Abstract])) OR (Acute Kidney Failure[Title/Abstract]))))
2. (((((((cardiac surgery[MeSH Terms]) OR (cardiac surgical procedures[Title/Abstract])) OR (cardiac operation[Title/Abstract])) OR (cardio surgery[Title/Abstract])) OR (cardiopulmonary bypass[Title/Abstract]))))
3. (((((((((pro-brain natriuretic peptide[MeSH Terms]) OR (N-terminal pro-BNP[Title/Abstract])) OR (proBNP(1-76[Title/Abstract]))) OR (NTproBNP[Title/Abstract])) OR (proBNP (1-76[Title/Abstract]))) OR (N-BNP peptide[Title/Abstract])) OR (NT-BNP[Title/Abstract])) OR (Amino-terminal pro-brain natriuretic peptide[Title/Abstract])) OR (aminoterminal pro-B-type natriuretic peptide[Title/Abstract])) OR (NT-proBNP[Title/Abstract])
4. #1 AND #2 AND #3

**Embase**

1. 'acute kidney injury network criteria':ti,ab,kw OR 'acute kidney injuries':ti,ab,kw OR 'kidney injuries, acute':ti,ab,kw OR 'kidney injury, acute':ti,ab,kw OR 'acute renal injury':ti,ab,kw OR 'acute renal injuries':ti,ab,kw OR 'renal injuries, acute':ti,ab,kw OR 'renal injury, acute':ti,ab,kw OR 'renal insufficiency, acute':ti,ab,kw OR 'acute renal insufficiencies':ti,ab,kw OR 'renal insufficiencies, acute':ti,ab,kw OR 'acute renal insufficiency':ti,ab,kw OR 'kidney insufficiency, acute':ti,ab,kw OR 'acute kidney insufficiencies':ti,ab,kw OR 'kidney insufficiencies, acute':ti,ab,kw OR 'acute kidney insufficiency':ti,ab,kw OR 'kidney failure, acute':ti,ab,kw OR 'acute kidney failures':ti,ab,kw OR 'kidney failures, acute':ti,ab,kw OR 'acute renal failure':ti,ab,kw OR 'acute renal failures':ti,ab,kw OR 'renal failures, acute':ti,ab,kw OR 'renal failure, acute':ti,ab,kw OR 'acute kidney failure':ti,ab,kw.
2. 'cardiac surgery':ti,ab,kw OR 'cardiac surgical procedures':ti,ab,kw OR 'cardiac operation':ti,ab,kw OR 'cardio surgery':ti,ab,kw OR 'cardiopulmonary bypass':ti,ab,kw.
3. 'pro-brain natriuretic peptide':ti,ab,kw OR 'n-terminal pro-bnp':ti,ab,kw OR 'probnp(1-76)':ti,ab,kw OR 'ntprobnp':ti,ab,kw OR 'n-bnp peptide':ti,ab,kw OR 'nt-bnp':ti,ab,kw OR 'amino-terminal pro-brain natriuretic peptide':ti,ab,kw OR 'aminoterminal pro-b-type natriuretic peptide':ti,ab,kw OR 'nt-probnp':ti,ab,kw.
4. #1 AND #2 AND #3

**Web of Science**

1. (((((((((((((((((((((((TS=(Acute Kidney Injury)) OR TS=(Acute Kidney Injuries)) OR TS=(Kidney Injuries, Acute)) OR TS=(Kidney Injury, Acute)) OR TS=(Acute Renal Injury)) OR TS=(Acute Renal Injuries)) OR TS=(Renal Injuries, Acute)) OR TS=(Renal Injury, Acute)) OR TS=(Renal Insufficiency, Acute)) OR TS=(acute Renal Insufficiencies)) OR TS=(Renal Insufficiencies, Acute)) OR TS=(Acute Renal Insufficiency)) OR TS=(Kidney Insufficiency, Acute)) OR TS=(Acute Kidney Insufficiencies)) OR TS=(Kidney Insufficiencies, Acute)) OR TS=(Acute Kidney Insufficiency)) OR TS=(Kidney Failure, Acute)) OR TS=(Acute Kidney Failures)) OR TS=(Kidney Failures, Acute)) OR TS=(Acute Renal Failure)) OR TS=(Acute Renal Failures)) OR TS=(Renal Failures, Acute)) OR TS=(Renal Failure, Acute)) OR TS=(Acute Kidney Failure)
2. ((((TS=(cardiac surgery)) OR TS=(cardiac surgical procedures)) OR TS=(cardiac operation)) OR TS=(cardio surgery)) OR TS=(cardiopulmonary bypass)
3. ((((((((TS=(pro-brain natriuretic peptide)) OR TS=(N-terminal pro-BNP)) OR TS=(NTproBNP)) OR TS=(N-BNP peptide)) OR TS=(NT-BNP)) OR TS=(Amino-terminal pro-brain natriuretic peptide)) OR TS=(aminoterminal pro-B-type natriuretic peptide)) OR TS=(NT-proBNP)) OR TS=(proBNP(1-76))
4. #1 AND #2 AND #3

**Cochrane Library**

1. MeSH descriptor: [Acute Kidney Injury] explode all trees
2. (Acute Kidney Injuries OR Kidney Injuries, Acute OR Kidney Injury, Acute OR Acute Renal Injury OR Acute Renal Injuries OR Renal Injuries, Acute OR Renal Injury, Acute OR Renal Insufficiency, Acute OR acute Renal Insufficiencies OR Renal Insufficiencies, Acute OR Acute Renal Insufficiency OR Kidney Insufficiency, Acute OR Acute Kidney Insufficiencies OR Kidney Insufficiencies, Acute OR Acute Kidney Insufficiency OR Kidney Failure, Acute OR Acute Kidney Failures OR Kidney Failures, Acute OR Acute Renal Failure OR Acute Renal Failures OR Renal Failures, Acute OR Renal Failure, Acute OR Acute Kidney Failure):ti,ab,kw
3. #1 OR #2
4. (pro-brain natriuretic peptide):ti,ab,kw OR (N-terminal pro-BNP):ti,ab,kw OR (NTproBNP):ti,ab,kw OR (N-BNP peptide):ti,ab,kw AND (NT-BNP):ti,ab,kw (Word variations have been searched) OR (Amino-terminal pro-brain natriuretic peptide):ti,ab,kw OR (aminoterminal pro-B-type natriuretic peptide):ti,ab,kw OR (NT-proBNP):ti,ab,kw
5. MeSH descriptor: [Thoracic Surgery] explode all trees
6. (cardiac surgical procedures):ti,ab,kw OR (cardiac operation):ti,ab,kw OR (cardio surgery):ti,ab,kw OR (cardiopulmonary bypass):ti,ab,kw
7. #5 OR #6
8. #3 AND #4 AND #7
