# Supplementary figures and images for: Diagnostic accuracy of NT-proBNP to predict the incidence of CSA-AKI: A systematic review and meta-analysis
Source: Medicine (Baltimore). 2024 Oct 25;103(43):e39479. doi: 10.1097/MD.0000000000039479 (PMC11521026; doi:10.1097/MD.0000000000039479)

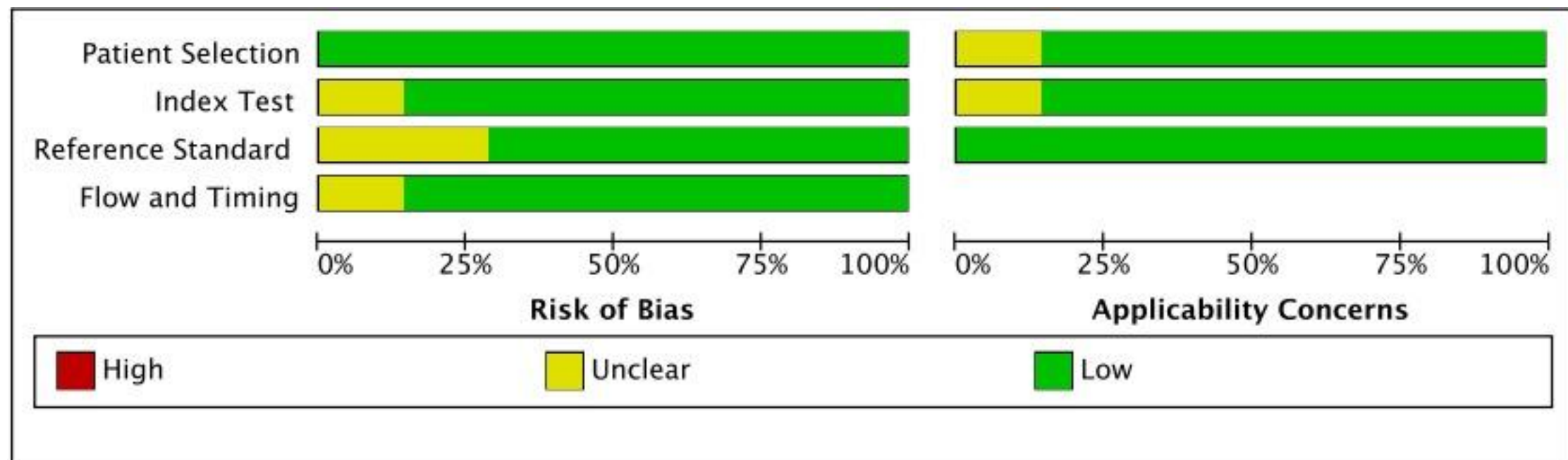

Supplement: Supplementary file 2 [file medi-103-e39479-s002.pdf]
